# Supplementary material for: Are carbon emissions trading and green financial instruments synergistic? -Comprehensive quantitative research based on content analysis
Source: PLoS One. 2024 Mar 7;19(3):e0298601. doi: 10.1371/journal.pone.0298601 (PMC10919601; doi:10.1371/journal.pone.0298601)
Supplement: S1 Data — (ZIP) [file pone.0298601.s001.zip › Data/地级市Z_F工作报告.zip!/║■─╧/╥μ╤⌠╩╨/└┤╫╘╣1⁄2╓┌║┼í╛┬φ┐╦╩2╛▌═°í┐_2011.docx]

各位代表：

　　现在，我代表市人民政府向大会作工作报告，并对《益阳市国民经济和社会发展第十二个五年规划纲要（草案）》作简要说明，请各位代表对政府工作报告和“十二五”规划《纲要（草案）》进行审议，并请市政协各位委员和其他列席同志提出意见。

　　2010年工作回顾：务实奋进，搭建了发展新平台

　　2010年是“十一五”的收官之年。一年来，我们在市委的正确领导下，团结带领全市人民，深入贯彻落实科学发展观，积极应对极为复杂的发展形势，攻坚克难，扎实工作，全面完成了市四届人大二次会议确定的各项目标任务。预计全年完成生产总值700亿元，比上年增长14.5%，其中第一、二、三产业增加值分别增长3.5%、21.3%和13.5%；完成财政总收入45.2亿元，同口径增长23.5%；完成固定资产投资437亿元，增长35%；实现社会消费品零售总额260亿元，增长18.5%；城镇居民人均可支配收入15389元，增长11.5%；农民人均纯收入5616元，增长13.7%。城镇登记失业率控制在4.2%以内。万元生产总值综合能耗下降4%，万元规模工业增加值能耗下降12%；化学需氧量和二氧化硫排放量完成省政府下达的目标。人口自然增长率为5.7‰。

　　一、加强产业建设，三次产业进一步发展

　　工业经济快速增长。预计全年实现工业增加值259亿元，比上年增长21.8%。其中规模工业增加值211亿元，增长24.4%；规模工业企业达到787家，比上年净增68家。完成工业投资237亿元，比上年增长42%，其中工业技改投资124亿元，增长51%。装备制造、食品加工、电力等十大产业实现增加值185.4亿元，增长23.8%。九大工业园区新引进项目155个，新开工项目146个，新建成投产项目97个，实现工业增加值110.6亿元，增长42.9%。高新技术产业实现增加值52.3亿元，增长35%。新增湖南名牌15个。加强对重点行业和重点用能企业的监管和节能改造，实施减排项目29个，淘汰落后产能企业47家。

　　农业经济稳步发展。预计全年实现农林牧渔业总产值230亿元，比上年增长3.5%。粮食连续7年实现增产，蔬菜、茶叶、棉花、生猪、水产品等产量和产值均有增长。农产品加工业实现增加值75亿元，比上年增长22.2%。新增农业产业化龙头企业38家。农业生产综合机械化水平达到54%，比上年提高2.9个百分点。农村土地流转工作稳步推进，共流转土地248万亩，其中耕地146万亩，占承包耕地总面积的40.2%；草尾镇农村土地信托流转试点工作取得成效。完成植树造林18万亩。防汛抗旱特别是防御山洪灾害取得胜利。成功举办了中国益阳首届水产品博览会暨大通湖大闸蟹美食旅游节，益阳被授予“中国淡水鱼都”称号。

　　第三产业逐渐上扬。旅游业较快发展，预计全年接待游客1296万人次，比上年增长14%；实现旅游总收入76.3亿元，增长21.5%。举办了中国益阳第三届洞庭渔火节、中国桃江第二届媒体定向越野赛。房地产业稳步健康发展，完成房地产开发投资60亿元，商品房销售金额37.3亿元，比上年分别增长15.4%和24.7%。“家电下乡”、“汽车摩托车下乡”、汽车和家电以旧换新活动成效明显，实现销售额16.5亿元。全年保险消费25亿元，比上年增长25%。对外贸易强势反弹，完成进出口总额3.5亿美元，增长55%，其中出口3.2亿美元，增长62%。

　　二、突出投资拉动，发展后劲进一步增强

　　项目建设扎实推进。组织开展“三项行动”，全市统一调度的120个重点项目完成投资135.9亿元，超额完成年度投资计划。桃花江核电站完成投资14.6亿元，白竹洲水电站4台机组并网发电。交通建设完成投资45.4亿元，比上年增长51.3%。二广高速桃马连接线一期、S308西姚公路等项目竣工通车；石长铁路复线益阳段、二广高速和杭瑞高速益阳段、沙头资江大桥、黄茅洲大桥等项目进展顺利；益娄高速、益马高速已举行开工动员；马溆高速、益南高速前期工作稳步推进；新建和改造农村公路1293公里；益阳至芦林潭千吨级航道益阳段整治、益阳港千吨级码头竣工投产。益阳东部新区完成基础设施建设投资4.5亿元。

　　引资争资成效明显。引进市域外资金203.6亿元，形成固定资产投资167.1亿元，比上年分别增长30.5%和26.7%。其中，引进境内省外资金111.9亿元，增长21.4%；直接利用外资10136万美元，增长10.3%。精心组织了“粤洽周”益阳承接汽车零配件产业转移专题招商对接会等招商活动，先后引进了39个汽车零配件项目、红联冷链项目等。立项争资力度加大。在中央预算内资金减少的情况下，精心挖掘项目，加大汇报衔接力度，共争取中央投资项目265个，投资金额10.8亿元。全年立项争资92亿元，比上年增长16.5%。

　　城镇建设步伐加快。大力实施“东进东接”战略，长株潭城市群“两型”社会示范区益阳东部新区改革建设总体方案已经省政府批准，完成了总体规划和土地利用规划调整。以创建国家卫生城市和省级文明城市为抓手，扎实推进城市建设。城市规划进一步完善。完成了《益阳城市特色研究》编制，启动了新一轮城市总体规划修编，完成了中心城区农贸市场等专项规划的编制。加强规划执法，中心城区四级联动共防共治违法违章建筑取得一定成效。城市基础设施建设加快推进。中心城区市政基础设施建设完成投资22.9亿元，完成了桃花仑路等14条主次干道和166条背街小巷的提质改造。完成了第二个“十万株树进城”计划。城市管理水平得到提高。在中心城区大力开展“创卫美家园”等主题活动，着力解决城市管理中的突出问题，特别是对道路保洁、渣土运输、户外广告等进行了集中整治，对砂石市场和河道采砂进行了根本治理，开展了烟花爆竹禁放工作，推行了街道精细化管理试点，市容市貌得到有效改善。小城镇建设扎实推进。全年完成小城镇建设投资15.9亿元，安化污水处理厂和沅江、桃江垃圾消纳场竣工运营。沧水铺、马迹塘、梅城等一批重点镇加快了建设步伐。

　　要素保障得到加强。年末金融机构人民币存贷款余额分别为606.4亿元和312.4亿元，比年初分别增长17.8%和17.1%；银企对接有效推进，继续开展“金融促发展，信贷早春行”活动，全市累计发放贷款109.6亿元；担保作用不断凸现，全年148家企业获得担保贷款7.5亿元；企业上市取得突破，汉森制药和太阳鸟游艇先后在深交所首发上市；小额贷款公司试点取得成效。用地保障不断加强，全年共批回建设用地1.28万亩，基本保证了重点项目建设的需求。出台了促进劳动力就地转移的政策措施，开展了“再就业援助月”等活动，举办各类招聘会120多场（次），较好地缓解了企业用工难问题。

　　三、坚持改革创新，体制机制进一步理顺

　　政府机构改革不断深化，市政府工作机构整合为32个，行政管理体制和运行机制进一步优化。公益性和经营性文化单位改革成效明显。医药卫生体制改革稳步推进，基本药物制度试点在赫山、桃江铺开。市直行政事业单位国有资产管理得到加强。公共资源交易平台建设经验在全省推广。事业单位人员聘用制度改革扎实推进。国企改革稳妥推进。财税管理体制不断完善，顺利完成了“省直管县”财政改革，国库集中支付制度逐步健全。税收协控联管成果得到巩固和发展。大通湖区统筹城乡发展试验区建设取得成效。集体林权制度改革的主体改革任务基本完成，配套改革正在加快推进。积极稳妥地推进资源价格和环保收费改革。

　　四、注重改善民生，社会建设水平进一步提高

　　就业再就业工作稳步推进。全市城镇新增就业3.64万人，新增农村劳动力转移就业8.29万人。社会保障水平不断提升。全市五大社会保险新增参保18万多人。基本医疗保障实现了城乡居民全覆盖，“新农保”新增沅江市为试点市，城乡低保应保尽保，救灾救济工作扎实有效。市流浪儿童保护中心投入使用。各项惠民政策得到落实。全市新增廉租住房9000套，城市和国有工矿棚户区改造顺利推进；完成农村2050户因灾倒房户的重建和3200户危房改造。解决农村饮水不安全人数24.79万人。新建和改扩建乡镇敬老院10所。9万农户参加政策性农业保险。对口扶贫工作有序推进。社会事业全面发展。着力推进科技进步，争取国家和省级科技计划750项，成立了益阳市科技成果转化服务中心。加快建设教育强市，进一步完善教育优先发展的政策措施，建成义务教育阶段合格学校57所，促进了教育公平和城乡教育均衡协调发展。医疗卫生事业健康发展，公共卫生服务体系建设得到加强，疾病预防控制工作成效突出，食品药品安全监管工作成效明显。爱国卫生工作有效开展，病媒生物防制工作进入全省先进行列。积极发展文化、体育事业，新建乡镇综合文化站17个、农家书屋459家，益阳小郁竹器制作技艺、南县地花鼓列入国家第三批非物质文化遗产保护名录；全民健身运动和竞技体育工作持续发展。人口计生基础工作不断加强，低生育水平持续稳定，保持了全省先进。生态建设进一步加强，创建省级生态示范村8个。注重抓好价格监测和市场监管工作，及时出台了稳定消费价格的措施。精神文明创建活动取得成效。大力弘扬“崇文尚义、通达超越”的城市精神，评比表彰了全市首届道德模范，市民素质和城市文明程度有新提高，创建省级文明城市通过考核验收。省级“双拥模范城”已获正式授牌。益阳市与以色列蒂科瓦市结为友好城市。社会大局和谐稳定。全面加强社会管理，社会治安防控体系逐步完善，社会治安综合治理居全省先进行列。进一步畅通信访渠道，着力化解矛盾纠纷，应对突发事件的能力不断增强。安全生产形势持续稳定，连续五年被评为全省先进单位。重视全民国防教育，国防动员、后备力量建设得到加强。人防工作全面发展，益阳荣获“全国人民防空先进单位”称号。广播电视、益阳日报、益阳门户网、统计、档案、保密、民族宗教、外事侨务、台办、科协、地方志、地震、消防、气象、水文、住房公积金、无线电管理、妇女儿童、老龄、残疾人、红十字会等工作取得新成绩。

　　五、坚持依法行政，政府自身建设进一步加强

　　依法行政能力不断增强。认真贯彻《行政许可法》和《湖南省行政程序规定》，政府科学民主依法决策机制逐步健全，行政执法责任制与过错追究制得到落实。“五五”普法圆满完成，“三调联动”工作取得成效。政府自觉接受人大及其常委会的法律监督和工作监督，自觉接受政协的民主监督，坚持重大事项向人大报告、向政协通报制度。认真听取人大代表、政协委员和各民主党派、工商联以及无党派人士的意见和建议，认真办理人大代表建议和政协委员提案，办复率100%。政务公开工作全面推进。市县两级政府部门政府信息公开指南目录编制率和发布率均达到100%。市政务中心部门进驻率达92%，项目进驻率达98.6%；区县（市）政务中心部门进驻率和项目进驻率均达80%以上；乡镇（街道）政务服务中心建成率达到59%。机关行政效能得到提高。扎实开展“创先争优”活动，全面加强公务员队伍建设，大力推行首问责任制、限时办结制、服务承诺制和领导干部问责制。绩效评估工作扎实推进。深化行政审批“两集中、两到位”改革，行政审批事项得到精简，审批流程明显优化。加强行政效能监察和审计监督，在全国地级市率先开通了“党风廉政舆情在线”，勤政廉政建设得到加强。

　　各位代表，过去一年的成绩来之不易。这是中共益阳市委正确领导的结果，是市人大、市政协支持和监督的结果，是全市人民团结拼搏的结果。在此，我代表市人民政府，向各位人大代表、政协委员，向奋斗在各条战线的广大干部群众，向离退休老同志，向各民主党派、工商联、无党派人士、各人民团体，向驻益人民解放军和武警官兵、公安干警，向中央和省属驻益单位，向所有关心、支持和参与益阳建设的各界朋友，表示衷心的感谢！

　　在肯定成绩的同时，我们也清醒地认识到面临的困难和问题。主要是：经济基础相对薄弱，总量规模较小；财政收支矛盾比较突出；农民持续增收难度较大；城市管理还存在薄弱环节；节能减排压力加大；政府工作人员办事效率有待进一步提高；社会不稳定因素依然存在。这些问题和不足，我们将高度重视，在今后的工作中采取有效措施认真加以解决。

　　2011年工作思路：科学谋划 把握发展新机遇

　　2011年是“十二五”的开局之年，是进一步应对复杂形势，继续保持经济平稳较快增长的关键之年，是新的挑战与机遇并存的一年。从全球看，世界经济有望继续恢复增长，但不稳定不确定因素仍然较多。国际金融危机影响深远，世界经济格局正在发生深刻复杂变化；从全国看，为了既保持经济平稳较快发展，又稳定价格总水平，国家实施积极的财政政策和稳健的货币政策，增强宏观调控的针对性、灵活性、有效性，对转变发展方式、调整经济结构提出了新的更高要求；从全市看，基础设施建设相对滞后，加快发展的基础尚不牢固，一些重点领域、关键环节的体制机制有待进一步理顺，涉及民生的深层次矛盾和问题凸显。但总体上看，我市加快发展仍然面临诸多有利因素。国家经济运行基本回归正常增长轨道，推动经济结构调整和发展方式转变迈出坚实步伐，沿海产业向中西部地区转移和劳动力就地转移的力度不断加大，长株潭“两型”社会试验区建设加快推进，益阳东接东进取得重大进展，环省会优势明显增强，全市广大干部群众加快发展的认识更加统一、氛围更加浓厚。因此，我们要正确研判形势，坚定信心，抢抓机遇，科学谋划，加快后发赶超步伐。

　　2011年政府工作的指导思想是：坚持以邓小平理论和“三个代表”重要思想为指导，全面贯彻落实党的十七届五中全会和中央、省委经济工作会议精神，按照省委推进“四化两型”的战略部署，紧紧围绕“坚持科学发展，奋力后发赶超，建设绿色益阳”的发展思路，进一步解放思想、开拓创新，切实优化经济结构，着力转变发展方式，大力推进新型工业化、新型城镇化、农业现代化和信息化，强力推进项目建设，切实抓好招商引资，加强城乡统筹，努力改善民生，确保经济平稳较快发展，确保人民群众幸福指数不断提高，确保社会和谐稳定。

　　2011年经济社会发展的预期目标是：生产总值增长13%，财政总收入增长18%，固定资产投资增长25%，社会消费品零售总额增长18%，规模工业增加值增长22%，城镇居民人均可支配收入增长12%，农民人均纯收入增长12%，城镇登记失业率控制在4.2%以内，万元生产总值综合能耗下降3%，化学需氧量、二氧化硫等主要污染物排放量比2010年分别削减2%，人口自然增长率控制在6.5‰以内。

　　实现上述目标，必须从益阳实际出发，突出工作重点，精心部署开展项目建设年等主题活动，以统筹兼顾、互动发展理念，科学谋划、加快启动一批重大产业建设、基础设施建设和民生改善的重点工程，推动产业集群式发展、城镇集约式扩张和农业现代化经营取得新的突破，打好“十二五”的开局仗。

　　2011年工作安排：乘势而上 创造发展新实绩

　　一、狠抓项目建设，进一步夯实发展基础

　　强力推进基础设施和重大产业项目建设。深入开展“三项行动”，把交通作为基础设施建设的重中之重，狠抓高速公路、铁路、国省干线等交通重点项目建设，实现交通建设的大投入、大发展。继续抓好石长铁路复线益阳段、G319益阳南线高速、G207干线公路等项目建设；推进长益常城际铁路、益马高速、益娄高速等项目征地拆迁和建设；争取马溆高速、益南高速等项目尽早开工建设。抓好一批重大产业项目建设，尤其要争取桃花江核电站尽快正式开工，争取安化海螺水泥项目开工建设；抓好三一工业园、益阳核电设备制造生产线、LED大功率半导体照明生产线、森华公司22万立方米板材生产线等续建项目。

　　大力优化项目建设环境。把“项目建设年”和优化发展环境活动结合起来抓，继续实施市级领导联系重点项目制度，加强对项目建设的考核；强化各级政府保障项目建设环境的责任，加大督查和问责力度；强化各部门服务项目建设的大局意识、责任意识，增强支持合力。切实解决项目建设中群众普遍反映的实际困难和问题，争取群众的理解、支持与配合。严厉打击强行参工参运、滋事阻工等违法行为，确保项目建设的顺利推进。

　　加大立项争资工作力度。认真研究国家加强和改善宏观调控、优化投资结构投资的方向和重点，围绕“三农”工作、改善民生和结构调整等重点领域，精心策划包装一批大项目、好项目，积极对接，争取中央和省里更多的投资。加强对市直部门立项争资目标任务的考核。

　　二、坚持工业强市，大力推进新型工业化

　　大力扶持重点产业和工业园区发展。抓住国家实施产业振兴的政策机遇，加快装备制造、电子信息、食品加工、竹木加工、新能源新材料等重点产业发展，构建具有益阳特色的新型工业体系。大力发展汽车零部件和船舶制造产业，全面提升我市装备制造业发展水平；加快电子信息产业聚集，壮大光伏产业、新型电子元件业和消费类电子整机业等电子产业集群；加快食品产业发展，打造食品产业知名品牌；大力培育战略性新兴产业；加快传统产业的改造升级。着力加强园区建设。进一步突出园区特色，完善园区功能，促进各类要素向园区集聚，增强园区产业承载能力，提高园区产出水平。做好园区规划调整工作。加快重点园区发展，特别是要强力推进益阳东部新区建设，全力支持益阳高新区申报国家级高新区。

　　扎实推进节能减排工作。把节能减排作为转方式、调结构和建设绿色益阳的重要抓手。加强对重点用能企业的监管，抓好重点行业、重点用能企业的节能改造，加强节能新技术的引进、研发和推广，实现万元规模工业增加值能耗下降2%的目标。大力实施污染减排行动，认真抓好污染减排项目，引导企业加强环保基础设施建设，淘汰落后产能，减少各类污染源，提高企业达标排放率。加强对区县（市）节能减排工作的考核，对没有完成目标任务的实行“区域限批”。

　　切实抓好招商引资。坚持诚信招商、实效招商、理性招商，探索建立专业招商工作机制，进一步创新招商方式，拓宽招商信息渠道，重点围绕我市比较优势产业，精心组织专业招商对接会，积极开展上门招商、小分队招商、以商招商、产业园区招商等活动，抓好产业带动力强的战略项目的引进，努力实现内联引资增长20%以上、直接利用外资增长10%以上的目标。狠抓投资环境建设，提高政务服务效率，加强全程代理工作，重视工业园区各项生活设施的配套，帮助外来投资企业解决项目开工、投产等方面的问题。加强区域交流与合作，提高对外开放水平。

　　三、突出城镇提质，加快推进新型城镇化

　　全面提升中心城区品位。抓好城市规划。基本完成城市总规修编工作，完善规划公示等制度，提高规划控制能力；充分发挥四级联动共防共治违章建筑机制的作用，坚持属地管理、以区为主，坚决遏制违法建设行为。加强城市建设。以创建国家卫生城市、国家森林城市为突破口，全力实施城市清洁工程、提质改造工程、绿化工程和亮化工程。重点抓好白马山路延伸、金花湖路等道路提质改造与新建、配套工程，全面完成背街小巷改造，启动资江五桥建设；实施好第三个“十万株树进城”工程，加大公园绿化建设力度全面完成中心城区农贸市场的标准化改造；推进第二水源建设。强化城市管理。进一步健全城市管理长效机制，重点抓好建筑垃圾的处置和户外广告管理，整治占道经营、占道停车，巩固砂石市场及河道采砂整治成果；完成主次干道环卫作业体制改革，加快环卫作业市场化进程；创新社区环卫管理制度，加强社区环卫设施建设；努力破解“城中村”治理难题。搞好城市经营。经营好城市各类资产特别是土地资产，实现土地价值的最大化。积极探索项目融资、信托融资、BT融资等融资模式，切实做好筹融资工作，为城市建设提供强有力的资金保障。

　　切实加快小城镇建设。按照发挥优势、突出特色、完善功能的原则，抓好小城镇的规划编制。进一步加快城镇基础设施和学校、卫生院、文化站、幼儿园等公共服务设施的建设，提高小城镇的综合承载能力和辐射带动作用。坚持产业兴镇，大力培育优势产业和特色经济，增强小城镇发展的后续动力。引导小城镇走经营城镇、市场运作、多元投资的道路，多渠道筹措小城镇建设资金。加快建设一批经济强镇、宜居小镇和文明卫生城镇，增强小城镇聚集人口、吸纳就业的能力。

　　扎实推进和谐社区建设。创新和完善社区体制机制，深化社区居民自治，不断提高社区管理和服务水平。大力培育社区社会组织，规范社区服务中心建设。进一步加大社区办公、服务基础设施建设力度，倡导“开放式办公、一站式服务”，积极推进政府公共服务覆盖社区，妥善解决社区居民就业、就学、就医等问题。深入开展“平安社区”、“和谐社区”创建活动，努力把社区建设成为管理有序、服务完善、文明祥和的社会生活共同体。

　　四、抓好城乡统筹，提高农业农村发展水平

　　进一步统筹城乡基础建设。通盘谋划城乡重大基础设施布局，实现城乡基础设施的共建共享和有效对接。加强农村公路网络建设。完成农村公路建设900公里。重点抓好S225平口至分水界等项目建设；做好S308柘溪至马路口、S206灰山港至宁益桥公路等项目的前期工作，争取早日动工建设；加快乡镇客运站场建设。加强农田水利建设。抓好人饮安全项目建设；高标准、高质量完成病险水库除险加固任务；建设高效节水的现代化灌排体系；认真实施洞庭湖综合治理近期项目，继续抓好大型排涝泵站更新改造、蓄洪安全和血防工程建设，加强重点流域治理和城市防洪工程建设；加强水土保持生态建设，抓好一批林业重点生态工程和小流域治理项目。加强农村通讯基础设施建设。启动新一轮农村电网改造升级工程。抓好新一轮新农村示范村、示范片建设。集中开展城乡环境卫生整洁行动，大力实施农村清洁工程，加强农业面源污染治理，改善农村生产生活条件。

　　加快发展现代农业。以促进农民增收为核心，大力发展现代农业。加快农村土地流转，继续完善和扩大土地信托流转试点，不断创新和完善土地流转模式，促进农业规模化、集约化经营。加强农产品基地建设，继续稳定粮食生产，深入开展粮棉油高产创建活动；大力发展蔬菜生产，抓好100万亩湘菜食材基地和一批特色蔬菜基地建设；大力发展黑茶生产，办好第二届黑茶文化节；进一步加快标准化规模健康养殖，加强生猪调出大县建设。落实动物疫病综合防控措施，防止重大疫情暴发流行。大力发展农产品精深加工，积极培育农产品加工龙头企业，提高农产品的附加值和市场竞争力。推进农民专业合作组织建设，提高农业生产的组织化程度。积极争取和落实农机购置补贴政策资金，提高农业机械化水平。不断完善农产品质量安全监管体系，提高农产品质量安全水平。加强农业科技创新体系建设，建设好市级农作物新品种区域试验基地和国家级农作物新品种展示基地，抓好超级稻示范推广等工作。扎实推进现代林业生态、林业产业和森林文化三大体系建设，提高现代林业示范市建设水平。

　　加强农村公共服务建设。统筹城乡公共服务，逐步实现城乡公共服务均等化。加强农村基础教育和农民素质教育，大力培育科技示范户、种养大户、农村经纪人等现代农业经营者。积极推进新型农村合作医疗制度，健全农村医疗卫生服务和医疗救助体系。加强乡镇文化站、村文化室等公共文化设施建设。抓好农村社会保障，切实改善困难群众基本生活。继续做好农村危房改造工作。认真落实农业补贴和农产品价格保护政策，深入开展涉农收费专项清理和农民负担重点治理。加大边远贫困地区的扶贫开发力度，继续做好移民后扶工作。

　　五、加快发展第三产业，努力扩大消费需求

　　大力发展旅游业。把旅游产业作为支柱产业来抓。以中心城区为核心，着力打造茶马古道文化旅游、南洞庭生态旅游等精品线路。推进旅游大项目和精品项目的开发建设，积极实施黄家湖、鱼形山、雪峰湖等景区规划及项目建设，进一步完善桃花江竹海、山乡巨变第一村、会龙山等景区的旅游设施，加大高等级酒店项目的建设力度，抓好市级游客服务中心等重点旅游配套设施建设。创新旅游宣传促销的手段和机制，不断开拓旅游市场。优化旅游业发展环境，规范旅游市场秩序。

　　积极发展服务业。加快研究制定促进服务业发展的政策措施。大力发展现代服务业，特别是加快发展现代物流、金融保险、电子商务、信息咨询等生产性服务业，着力抓好益阳物流园、蔬菜物流园和兰溪现代粮食产业园建设。加快益阳东部新区高端三产业区的建设，打造现代服务业发展的新高地。规范发展会计、法律、经纪等中介服务。提高餐饮住宿、商贸流通、交通运输、社区服务等传统服务业的质量水平。

　　努力扩大城乡消费。全面落实国家扩大消费的各项政策。千方百计增加城乡居民收入特别是低收入群体的收入，提高居民消费能力。发挥供销、邮政等流通主渠道作用，充分利用“万村千乡市场工程”网络，建立健全农村流通网、连锁经营网和物流配送体系，努力拓展农村消费。着力培育和扩大消费热点，促进房地产和汽车市场健康发展，引导扩大文化、旅游、保健等消费。整顿和规范市场秩序，加强消费维权，优化消费环境。

　　六、坚持以人为本，加快发展民生事业

　　全面发展社会事业。深入实施教育强市战略。切实加大教育投入，确保预算内教育经费占财政支出的比例同口径提高2个百分点；优化教育资源配置，推进合格学校建设，促进教育公平和城乡教育均衡发展；深化教育教学改革，全面实施素质教育，加强职业教育基础能力建设；着力提高教师队伍整体素质。加强文化基础设施建设，提高公共文化服务水平，抓好文化遗产保护和文化资源的开发利用，加快文化产业发展。加强全民健身体系建设，组织承办好湖南省第五届农民运动会，进一步增强竞技体育竞争力。加强公共卫生服务体系建设，抓好医疗卫生项目建设，重视妇幼保健和中医事业发展，切实加强重大疾病防控、卫生应急和医疗监审工作。扎实抓好人口计生工作，加大遏制违法生育力度，稳定低生育水平。高度重视环境保护，突出对重点流域、区域和行业的环境整治，促进环境质量持续改善。加快气象防灾减灾体系建设。支持工、青、妇等群团组织依法依规参与社会管理与公共服务，抓好新一轮妇女儿童发展规划。继续做好统计、档案、保密、民族宗教、外事侨务、台办、科协、残疾人、地方志、地震、人防、水文、住房公积金、无线电管理、红十字会等工作。

　　切实加强精神文明建设。深化社会主义核心价值体系教育，提高市民素质。继续开展道德模范评选和宣传学习活动。巩固省级文明城市创建成果。大力推广创“五星村、十星户”经验，抓好农村精神文明建设工作。开展文化环境专项整治，净化互联网、媒体和出版物市场。健全未成年人思想道德建设网络，加强校园周边环境综合治理，努力营造未成年人健康成长的良好社会环境。进一步办好《益阳日报》、益阳电视台、益阳广播电台、益阳新闻网和益阳门户网站。

　　抓好就业再就业工作。加强就业技能培训和职业教育，提高劳动力素质。加强公共就业服务，落实促进劳动力就地转移就业的各项措施，开展多种形式的就业援助，为劳动力就业和企业用工提供优质服务。优化就业环境，督促各类企业全面实施劳动合同制度，加快建立企业工资集体协商和企业集体合同制度，加强劳动监察执法，构建和谐劳动关系，维护劳动者权益。

　　不断完善社会保障体系。进一步强化社保扩面征缴，稳步提高社会保障水平。认真做好赫山区、沅江市“新农保”试点工作，扩大城镇居民养老保险覆盖面，巩固和发展城镇居民医疗保险，大力发展失业、工伤和生育保险。拓宽保险服务领域，促进保险业快速发展。抓好城乡社会救助体系建设，提高城乡低保保障标准，发展慈善事业，抓好救灾救济和价格临时补贴工作。积极争取国家专项补助资金和多渠道自筹资金，切实加强廉租房建设，规范廉租房分配，大力发展公共租赁住房，继续实施城镇住房困难家庭租赁补贴与租金核减政策，加快推进棚户区改造，逐步解决中低收入居民家庭住房困难问题。加快社会福利事业发展，启动中心城区老年公寓项目。落实优抚标准自然增长机制，继续做好乡镇敬老院改扩建工作，提高农村五保老人集中供养率和供养水平。

　　努力维护社会和谐稳定。严格落实综治、维稳工作责任，全面推行社会稳定风险评估机制，完善“三调联动”工作机制，开展经常性的矛盾纠纷排查调处，建立健全以群众工作统揽信访工作的体制机制，加强信访大厅建设，做好群众来信来访工作，有效预防和化解群体性事件。强化综治基层基础工作，强力推进村（社区）为主的综治、维稳工作模式，抓好城市治安电子防控体系建设，全面实施流动人口居住证制度，加强专业群防群治队伍建设，开展治安重点整治专项行动，维护良好的社会治安秩序。强化企业安全生产主体责任和各级政府的监管责任，深入开展安全隐患排查治理和安全执法，加强安全生产基层基础工作，有效预防和减少生产安全事故。加强食品药品监管，确保人民群众食品和用药安全。抓好应急救援工作，提高应急救援能力。加强国防和后备力量建设，支持武警、消防部队建设。

　　七、推进改革创新，不断增强发展活力

　　注重抓好要素保障。继续开展“金融促发展，信贷早春行”活动，不断扩大信贷规模；积极推进商业银行分支机构和总分制村镇银行筹建工作；进一步落实小额贷款公司试点政策，推动区县（市）小额贷款公司试点扩面；扎实抓好企业上市工作，推动艾华电子、克明面业、湘运集团等企业加快改制上市步伐；扩大企业私募股权融资规模；优化金融生态环境，加强社会信用体系建设，打击非法集资等金融违法行为，健全金融风险防范和化解机制。全面落实国家鼓励民间投资的政策，千方百计激活民间投资。规范建设用地管理，重点保证市级以上重点建设项目和工业项目用地，优先安排民生工程、基础设施、社会事业等项目用地；严格耕地保护，实施好环洞庭湖基本农田重大建设项目。加快创新体系建设，促进产学研结合，充分发挥市科技成果转化服务中心和专利交易中心的作用，为传统产业技术改造和结构升级提供科技支持。加强人力资源开发，统筹推进各类人才队伍建设，促进人才总量增长和结构优化。加快海关、商检等平台建设，扶持重点出口企业发展，进一步扩大对外贸易。

　　积极推进各项改革。深化机关事业单位工资收入分配制度改革。稳步推进财政体制改革，巩固“省直管县”财政体制改革成果，深化国库集中支付改革，不断完善基层财政管理体制。继续加大税收协控联管工作力度。深化国有企业和国有资产管理改革，健全国有资产监管体系。深化资源环境价格改革，促进资源节约和环境保护。深化集体林权制度改革，建立现代林业产权制度。深化医药卫生体制改革，继续推进基本药物制度，探索开展公立医院改革试点工作。全面深化文化体制改革。积极推进教育管理体制和教师人事制度改革。

　　大力推动全民创业。全面落实国家关于鼓励创业的优惠政策，进一步完善支持创业的财税、信贷等扶持政策。加大全民创业培训力度。加快建设创业园区或创业基地，引导和支持城镇下岗失业人员、进城农民工、大中专院校毕业生、退伍军人和有劳动能力的残疾人自主创业。畅通创业项目行政审批绿色通道，完善创业社会化服务体系，优化创业环境。大力发展非公有制经济，充分发挥非公有制企业在全民创业中的示范、带动作用。注重发挥商协会在市场经济建设中的组织作用。

　　深入建设效能政府、法治政府和廉洁政府。强化民主、法治、责任、服务等理念，加快建设现代政府。进一步转变政府职能，巩固和扩大政府机构改革成果。深入开展政府绩效评估工作，推进政府管理创新，降低行政成本，提高行政效能。贯彻实施《行政许可法》，深入推进行政审批制度改革，继续精简和调整行政审批事项，优化行政审批流程。严格执行《湖南省行政程序规定》，加强行政执法监督，进一步规范自由裁量权。加强政务服务中心建设，完善政务公开和办事公开制度，加快电子政务建设。全面启动“六五”普法。严格依法行政，认真执行人大及其常委会的决议、决定，自觉接受人大的法律监督、工作监督，主动接受政协的民主监督，定期向人大报告工作和向政协通报情况，广泛听取各民主党派、工商联和无党派人士的意见。加强对重要部门、重点领域和关键环节的行政监察和审计监督，强化“党风廉政舆论在线”网络监督，继续加强公共资源交易中心建设，健全和落实行政问责制及相关配套制度，促进政府各部门正确履行职责，强化清正廉洁的政风。

　　“十二五”规划《纲要（草案）》的说明：

　　奋力后发赶超 实现发展新突破

　　中共益阳市委四届五次全会通过的《中共益阳市委关于制定益阳市国民经济和社会发展第十二个五年规划的建议》，提出了未来五年益阳市国民经济和社会发展的指导思想、发展战略和目标任务。根据《建议》精神，市政府制定了《益阳市国民经济和社会发展第十二个五年规划纲要（草案）》，已提请大会审议。下面，就几个问题作简要说明。

　　一、《纲要（草案）》的编制过程

　　市委、市政府对制定“十二五“规划工作高度重视。早在2009年10月，市委、市政府就开始部署相关准备工作。2009年12月，“十二五”规划领导小组成员会议讨论确定了7个研究课题和33个专项规划，并明确了责任单位；到2010年9月，所有研究课题和专项规划全面完成，为编制《纲要（草案）》奠定了基础。从2010年10月开始，组织相关部门和专家召开调研座谈会，对工业、农业、经济合作、城乡建设等课题进行了集中研究，并先后到各区县（市）开展了“十二五”规划专题调研，为《纲要（草案）》的编制和完善提供了重要支撑。

　　规划《纲要》初稿形成后，市委、市政府在全市范围内开展了多种形式的征求意见活动。先后向市四届人大常委会第16次会议、市四届政协第26次主席会议征求意见，向市直单位和各区县（市）征求意见，组织市专家联合会各个行业的专家开展专业咨询，在益阳门户网站上向广大市民征求意见。《纲要（草案）》充分吸纳了各方面的合理意见与建议，体现了科学办规划、开门办规划的要求。《纲要（草案）》的编制过程，是发扬民主、集思广益、科学决策的过程，是动员社会力量、凝聚社会智慧、形成社会共识的过程。

　　二、《纲要（草案）》对发展基础的分析判断

　　过去的五年，是我市发展进程中非常重要的五年，是开拓进取、跨越发展的五年。我们坚持以科学发展观为统领，全力实施“工业强市、项目立市、开放活市、科教兴市、依法治市”五大战略，突出抓好招商引资和项目建设，努力推进“三化”进程，协调发展社会事业，有效应对金融危机和自然灾害，经济社会发展取得了较大成就，全面完成和超额完成了“十一五”规划的各项目标，为“十二五”经济社会发展打下了良好基础。

　　五年来，我市综合实力实现新跨越。生产总值年均增长13.5%，财政总收入年均增长24.2%，全社会固定资产投资年均增长38.9%。新型工业化进程加快，产业结构不断优化，高新技术产业快速发展。城乡统筹发展稳步推进，以现代农业建设为标志的农村经济加速发展。基础产业和基础设施建设成效显著，在水利、能源、交通等领域建成或新开工一大批重大工程。

　　五年来，我市改革开放取得新成效。农村、医疗等重要领域和关键环节的改革取得重大突破。区域发展迈出新的步伐，创建益阳东部新区，并纳入长株潭“两型社会”试验区大河西先导区。对外开放水平稳步提高，引进战略投资者、承接产业转移取得实效。

　　五年来，我市民生改善取得新进展。人民生活水平不断提高，城镇居民人均可支配收入和农民人均纯收入年均增长13.3%和13.2%。五年累计新增城镇就业20.3万人，城镇登记失业率控制在4.3%以内，实现农村劳动力新增转移就业37万人，社会保障水平进一步提高。各项社会事业加快发展。

　　五年来，我市政治建设、文化建设、社会建设等都取得了较大的发展，呈现出崭新面貌。

　　三、《纲要（草案）》对发展战略和发展目标的设定

　　“十二五”时期是我市经济加快发展、社会逐步转型的关键时期。国际国内环境总体向好，省内市内有利条件较多，但也存在不少制约因素和困难。

　　《纲要（草案）》贯穿了科学发展的主题，体现了市委《建议》提出的指导思想，这就是：以邓小平理论和“三个代表”重要思想为指导，全面贯彻落实科学发展观，按照省委“四化两型”总体战略，紧紧围绕“坚持科学发展、奋力后发赶超、建设绿色益阳”的总体思路，把新型工业化作为后发赶超的第一推动力，把招商引资作为富民强市的第一选择，把人民群众满意作为检验发展成效的第一标准，全面实施工业强市、绿色发展、开放带动和城乡统筹发展战略，加速推进新型工业化、新型城镇化、农业现代化，着力扩总量、转方式、调结构、惠民生，为建设经济繁荣、社会进步、生态优美、人民幸福的新益阳而奋斗。

　　根据市委《建议》确定的指导思想，《纲要（草案）》提出了“十二五”时期益阳经济社会发展的主要目标，即：经济发展实现新跨越，绿色益阳建设取得新成效，改革开放取得新突破，人民生活水平得到新提升。具体指标是：“十二五”期间，全市生产总值年均增长13%，财政总收入年均增长20%，固定资产投资年均增长25%。这几个指标均略低于“十一五”时期的增速，主要是为了让我们腾出更多的时间和空间去发展社会事业，改善民生，节约资源和保护环境，推进城乡协调发展。《纲要（草案）》同时提出，“十二五”期间，城镇居民人均可支配收入、农民人均纯收入年均分别增长12%，资源环境方面的约束性指标从严控制，确保完成省定目标。我们将社会发展和民生、环境等指标定得略高一些，这就要求我们更加重视民生，进一步抓好科学发展、统筹协调发展。

　　四、《纲要（草案）》对发展重点和发展措施的强调

　　《纲要（草案）》对“十二五”时期的经济建设、政治建设、文化建设、社会建设等方面，都作出了全面部署，提出了明确的任务和措施。

　　一是以新型工业化为第一推动力，加快后发赶超步伐。《纲要（草案）》提出，要继续实施“工业强市”战略，推动产业结构调整，促进经济结构的优化升级、财政收入的快速增长、经济实力的显著增强。重点任务是：加快构建益阳新兴工业体系，积极支持企业加快发展，推动产业向园区集聚，增强自主创新能力，加速推进信息化与工业化的融合。在推进新型工业化的过程中，尤其要注重促进发展方式转变，大力发展“两型产业”，加大节能减排力度，加强生态建设，着力建设绿色益阳。

　　二是加快推进新型城镇化，统筹城乡区域协调发展。打破城乡二元结构、实现城乡和区域协调发展，是新时期经济发展的战略任务。《纲要（草案）》根据区域发展的不同状况，进行了分类部署，提出要构建科学合理的城镇体系，做大做强中心城区，完善城镇功能配套，统筹城乡和区域发展。要进一步实施“东接东进”，大力推进东部新区建设，加快建成“两型”社会建设示范区。要抓好中心城区、县城、重点乡镇、中心村等环节，在遵循城乡发展建设差别性和互补性的基础上，统筹资源，优化配置，推进城乡一体化。

　　三是加快推进农业现代化，全面加强新农村建设。要以规模经营和提质增效为基本要求，加快农村土地流转，建设优势农产品基地，大力发展农产品加工业，建立健全农村服务体系，着力培育农业新兴业态，不断调优调强农业产业结构，提高产业化经营水平，进一步巩固农业基础地位。要加快推进新农村建设，着力抓好规划编制、基础设施建设、村容村貌治理、工业布局、乡风文明建设、农村民主管理和新农村示范村建设等工作。

　　四是加快发展现代服务业，不断扩大消费需求、促进消费升级。现代服务业的发展繁荣是产业结构优化的重要体现。《纲要（草案）》结合我市实际，提出要加快发展旅游业，要以服务外包业、现代物流业、中介服务业为重点，大力发展生产性服务业，要以餐饮住宿业、商贸零售业、社区服务业、房地产业为重点，全面提升生活性服务业。

　　五是加强基础设施建设，进一步夯实发展基础。《纲要（草案）》强调，要坚持“统筹规划、适度超前”的原则，进一步加强基础设施建设及相应的功能配套建设。重点突出交通、水利、能源和信息基础设施建设，到2015年，基本建成益阳城区交通主枢纽，安化东坪镇、桃江马迹塘镇、南县南洲镇交通次枢纽，全面打通对外通道，通畅内部交通，同时使电网设施更加完善，防洪抗旱减灾能力进一步提高，水资源保护进一步加强。

　　六是促进发展方式转变，着力建设绿色益阳。建设绿色益阳是我们正在实施的重大战略。《纲要（草案）》提出，要加快“两型”社会建设，坚持节约、综合利用能源和资源，促进工农业清洁生产，减少污染物排放；坚持生态保护与污染防治并重，加强生态环境建设，使人口增长与社会生产力发展相适应，经济建设与资源、环境相协调，实现社会进步良性循环。要通过大力发展“两型产业”、加大节能减排力度、加强生态建设，确保“十二五”期间全面完成省定节能减排目标任务。

　　七是实施民生工程“六大行动计划”，着力改善民生民利。《纲要（草案）》切实关注人民群众最关心、最直接、最现实的增收、就业、社保、医疗、社会救助等问题，提出要重点实施好增收富民、促进就业、社保救助、教育均衡、人口和健康、文化普惠等“六大行动计划”，不断完善公共服务体系，着力改善民生民利，全面推进社会事业发展。

　　八是深化改革和扩大开放，增强经济社会发展活力。当前，我市生产力总体水平还不高，影响生产力发展的深层次矛盾和体制性障碍还不少，必须坚定不移地推进改革开放，最大限度地解放和发展生产力。《纲要（草案）》就如何深化体制机制改革和继续扩大对内对外开放作了全面部署。

　　同时，《纲要（草案）》还对创新社会管理、切实加强作风建设、推进民主政治建设、努力构建和谐益阳做出了具体部署。

　　各位代表！全面完成今年和“十二五”期间的任务，对我们提出了新的更高的要求。站在新的起点上，我们责任重大、使命光荣。让我们在中共益阳市委的坚强领导下，解放思想，开拓创新，同心同德，扎实工作，为抓好“十二五”起步发展，开创益阳后发赶超新局面而努力奋斗！
